# A cluster randomized controlled trial for a multi-level, clinic-based smoking cessation program with women in Appalachian communities: study protocol for the “Break Free” program

**DOI:** 10.1186/s13722-022-00295-5

**Published:** 2022-02-14

**Authors:** Joanne G. Patterson, Tia N. Borger, Jessica L. Burris, Mark Conaway, Robert Klesges, Amie Ashcraft, Lindsay Hauser, Connie Clark, Lauren Wright, Sarah Cooper, Merry C. Smith, Mark Dignan, Stephenie Kennedy-Rea, Electra D. Paskett, Roger Anderson, Amy K. Ferketich

**Affiliations:** 1 Division of Epidemiology, College of Public Health, The Ohio State University, 354 Cunz Hall, 1841 Neil Avenue, Columbus, OH 43210 USA; 2 The Ohio State University Comprehensive Cancer Center, Columbus, OH USA; 3 Department of Psychology, University of Kentucky, Lexington, KY USA; 4 Department of Public Health Sciences, University of Virginia, Charlottesville, VA USA; 5 West Virginia University, Morgantown, WV USA; 6 UVA Cancer Center, University of Virginia, Charlottesville, VA USA; 7 College of Public Health, The Ohio State University, Columbus, OH USA; 8 Department of Internal Medicine, College of Medicine, University of Kentucky, Lexington, KY USA; 9 Markey Cancer Center, University of Kentucky, Lexington, KY USA; 10 West Virginia University Cancer Institute, Morgantown, WV USA; 11 Department of Medicine, School of Medicine, West Virginia University, Morgantown, WV USA; 12 Division of Cancer Prevention and Control, Department of Internal Medicine, College of Medicine, The Ohio State University, Columbus, OH USA; 13 School of Medicine, University of Virginia, Charlottesville, VA USA

**Keywords:** Smoking cessation, Rural health, Cervical cancer prevention, Implementation science, Clinic-based interventions

## Abstract

**Background:**

The cervical cancer burden is high among women living in Appalachia. Cigarette smoking, a cervical cancer risk factor, is also highly prevalent in this population. This project aims to increase smoking cessation among women living in Appalachia by embedding a smoking cessation program within a larger, integrated cervical cancer prevention program.

**Methods:**

The broader program, the *Take CARE* study, is a multi-site research collaborative designed to address three risk factors for cervical cancer incidence and mortality: tobacco use, human papillomavirus (HPV) infection, and cervical cancer screening. *Break Free* is a primary care clinic-based implementation program that aims to promote smoking cessation among female smokers in Appalachia by standardizing clinical practice protocols. *Break Free* includes: (1) implementation of a tobacco user identification system in the Electronic Health Record, (2) clinic staff and provider training on the Ask, Advise and Refer (AAR) model, (3) provider implementation of AAR to identify and treat women who want to quit smoking within the next 6 months, (4) facilitated access to cessation phone counseling plus pharmacotherapy, and (5) the bundling of *Break Free* tobacco cessation with HPV vaccination and cervical cancer screening interventions in an integrated approach to cervical cancer prevention. The study spans 35 Appalachian health clinics across 10 healthcare systems. We aim to enroll 51 adult female smokers per health system (total N = 510). Baseline and follow-up data will be obtained from participant (provider and patient) surveys. The primary outcome is self-reported 12-month point prevalence abstinence among enrolled patients. All randomized patients are asked to complete follow-up surveys, regardless of whether they participated in tobacco treatment. Data analysis of the primary aims will follow intent-to-treat methodology. Secondary outcomes will assess program implementation and cost effectiveness.

**Discussion:**

Addressing high tobacco use rates is critical for reducing cervical cancer morbidity and mortality among women living in Appalachia. This study evaluates the implementation and effectiveness of a smoking cessation program in increasing smoking cessation among female smokers. If results demonstrate effectiveness and sustainability, implementation of this program into other health care clinics could reduce both rates of smoking and cervical cancer.

*Trial registration* NCT04340531 (April 9, 2020)

**Supplementary Information:**

The online version contains supplementary material available at 10.1186/s13722-022-00295-5.

## Background

Appalachia—a mountainous region that spans 13 states and is populated by approximately 35.7 million individuals [1]—has a higher burden of cancer than other regions in the United States (US). The cancer mortality rate in Appalachia is 10% higher than the national rate, and tobacco-related cancers are highly prevalent in this region [2–4]. Cervical cancer, a preventable condition, is 23% more prevalent in Appalachian regions and women are 25% more likely to die from cervical cancer in this region than their non-Appalachian counterparts [2–4]. These regional disparities are alarming as national incidence rates for cervical cancer have consistently been declining [5] with the growth of initiatives to address modifiable risk factors (e.g., smoking, human papillomavirus (HPV) infection rates, access to cervical cancer screening). In contrast, women in Appalachia continue to experience disparities across a multiplicity of cervical cancer risk factors [6] including disparately high smoking rates [7], low uptake of HPV vaccination [8], and low rates of cervical cancer screening [9]. To reduce cervical cancer morbidity and mortality in Appalachia, implementation research is needed to test the effectiveness and sustainability of evidence-based interventions that address cervical cancer risk factors.

Cigarette smoking is a modifiable risk factor for cervical cancer. Studies from the US, Europe, and China indicate that female smokers are at increased risk of cervical cancer and/or pre-cancerous lesions [10–12]. Smoking prevalence rates in Appalachian communities are elevated; for example, in Appalachian Ohio, 23–44% of adults report current smoking [13] and in Appalachian Kentucky current smoking prevalence is 33% rate [14]; over twice the national rate [15]. Multilevel barriers to smoking cessation exist in Appalachia. Structural determinants (e.g., high poverty rates and low educational attainment), pro-tobacco social norms, underinvestment in smoking prevention, and limited access to cessation treatment drive cessation disparities at the population level [16–18]. However, individual-level determinants including high perceived stress, cessation ambivalence, and distrust in pharmacotherapy may be additional barriers to smoking cessation in this population [19–21]. Identifying culturally acceptable, effective, and scalable interventions is critical for increasing cessation among women living in Appalachia. Undoubtedly, such interventions could have a significant, positive impact on reducing cervical cancer disparities among women living in Appalachia, in addition to the many tobacco-related chronic diseases that are widespread in this region [22].

The US Public Health Service *Clinical Practice Guideline, Treating Tobacco Use and Dependence* [23], recommends that all healthcare systems and providers systematically assess and document tobacco use, adopt motivational techniques to encourage quitting, and provide evidence-based tobacco cessation. Healthcare providers can be effective advocates for smoking cessation, with brief advice alone increasing the odds of quit attempts and abstinence [24]. However, only 50–75% of providers regularly screen patients for tobacco [25–28] and just over half of smokers report being advised to quit smoking by their providers [27–29]. As such, developing clinic-based interventions that increase the rate at which smokers are identified, advised to quit, and provided or referred for cessation treatment is critical for eliminating tobacco-related health disparities among women living in Appalachia.

Primary care clinics are an ideal setting to reach and engage women in smoking cessation treatment, as more than 70% of smokers visit a provider each year [30]. Implementing the *Clinical Practice Guideline* can be challenging, however, for primary care practices and providers, due to lack of training, limited knowledge about the recommendations, lack of patient motivation to quit, and inadequate billing and reimbursement systems [31–34]. In Appalachia, these challenges are compounded by limited access to comprehensive cessation programs that offer sustained assistance, inadequate patient education resources, and practical barriers to follow-up [35]. Barriers notwithstanding, training that targets providers’ knowledge, attitudes, and practice behaviors can increase adherence to the *Clinical Practice Guideline* [23] and increase smoking abstinence at the patient level [24]. By giving providers tools to change clinical practice behaviors, implementing provider-targeted educational interventions may be an effective strategy for reducing smoking among women living in Appalachia.

To address the high rates of both cervical cancer and smoking in Appalachian women, a transdisciplinary team of investigators developed the *Take CARE* (Clinical Avenues to Reach Health Equity) study, a multi-site research collaborative comprising four universities and participating clinics located in Appalachian counties of Kentucky, Ohio, Virginia, and West Virginia. *Take CARE* is designed to address the burden of cervical cancer incidence and mortality in Appalachia through the delivery of an integrated clinic-based cancer prevention program. Funded by the National Institutes of Health (P01CA229143), the *Take CARE* study focuses on the major causes of cervical cancer including: tobacco smoking, HPV infection, and lack of cervical cancer screening. Research in this collaborative spans both qualitative and experimental designs and involves community participation and engagement. Study endpoints include measures of intervention implementation, quality improvement processes, and client outcomes. This paper describes the study protocol for *Break Free*, one of three initiatives in the *Take CARE* study (ClinicalTrials.gov ID: NCT04340531). *Break Free* is an evidence-based smoking cessation intervention designed to address the lack of provider-led tobacco assessment and intervention in Appalachian healthcare clinics and, ultimately, increase smoking cessation rates among women living in Appalachia. The aims of this study are to determine: (1) effectiveness of the *Break Free* intervention, (2) satisfaction with the intervention, and (3) sustainability of the intervention.

## Methods

This protocol (Version 3, 11/23/2021) has been written according to the recommendations of the SPIRIT 2013 statement, a guideline that defines the standard elements of a protocol (see Additional file 1). Any modifications to the protocol are discussed during biweekly research team meetings and then communicated to the *Take CARE* Steering Committee, Institutional Review Board (IRB), trial registries, and participants as needed by IRB-approved study team members.

### Study overview

We are conducting a Type III hybrid effectiveness-implementation study to evaluate a multilevel smoking cessation program for women in Appalachia. Type III hybrid effectiveness-implementation study designs focus on assessing implementation outcomes while also assessing clinical outcomes associated with study implementation. Our primary study goal is to test the implementation of a clinic-based, supported tobacco cessation program in Appalachia, using measures of adoption of and fidelity to the intervention. However, we are also measuring patient-level effects of the intervention on smoking cessation rates. The evaluation will occur over 5 years and includes health system-, provider- and patient-level interventions for participating clinics from 10 health systems. The multilevel program components consist of recommendations from the *Clinical Practice Guideline* [23]. These include recommendations that health systems implement a tobacco user identification system at the clinic level (ask about, and document, smoking status) and providers advise all smokers to quit and refer those ready to quit to *Break Free* phone counseling, which provides supported smoking cessation. Notably, *Break Free* phone counseling provides treatment to women who plan to quit within six months, allowing the content to be tailored to the level of readiness to quit for each individual participant.

### Break Free

*Break Free* was developed through our prior work with health systems in Appalachian regions of Virginia [36] and Ohio [26, 37–39] and modified by the study team to have broad applicability across diverse primary care settings. *Break Free* is one of three initiatives in the *Take CARE* program. This overarching program also includes four cores (shared resources): Administrative, Survey and Data Collection, Intervention and Consortium, and Biostatistics and Evaluation. The Administrative Core supports the *Take CARE* program by providing leadership in program planning and development, implementing communication channels, and facilitating integration of the *Take CARE* Program cores and the three research projects. The Survey and Data Collection Core will assist initiatives with their data collection activities, develop a database of indicators and measures to be applied across all three research projects, and provide support for interactions with clinic electronic health systems and data capture. The Intervention and Consortium Core will lead efforts to develop and deliver the interventions being tested in each initiative, and maintain communications with community partners. Finally, the Biostatistics and Evaluation Core will provide investigators with a centralized resource to plan, implement, monitor, and analyze data from all three initiatives and the overall *Take CARE* program, including cost-effectiveness. Information on how each of the *Take CARE* cores will support *Break Free* project planning, implementation, data collection and analysis is described below in the context of study design, data collection, analysis, and dissemination.

Prior to launching the *Take CARE* program, lead study investigators proactively engaged executive leadership from each health system to obtain their commitment to *Take CARE* and its three initiatives, including *Break Free*. Throughout each phase of the study, lead investigators will maintain contact with health system leadership via regular communication with medical directors of participating clinics/health systems. At the end of the study, we will also present overall results from *Take CARE*, including study-specific results from *Break Free*, to the boards of participating health systems. Each initiative in *Take CARE* is also supported by a Community Advisory Board comprised of leaders from diverse community-based institutions. This Board will inform best practices for project implementation, including issues of access and barriers to uptake, as well as methods for ensuring sustainability beyond the grant funding period.

**Fig. 1:**
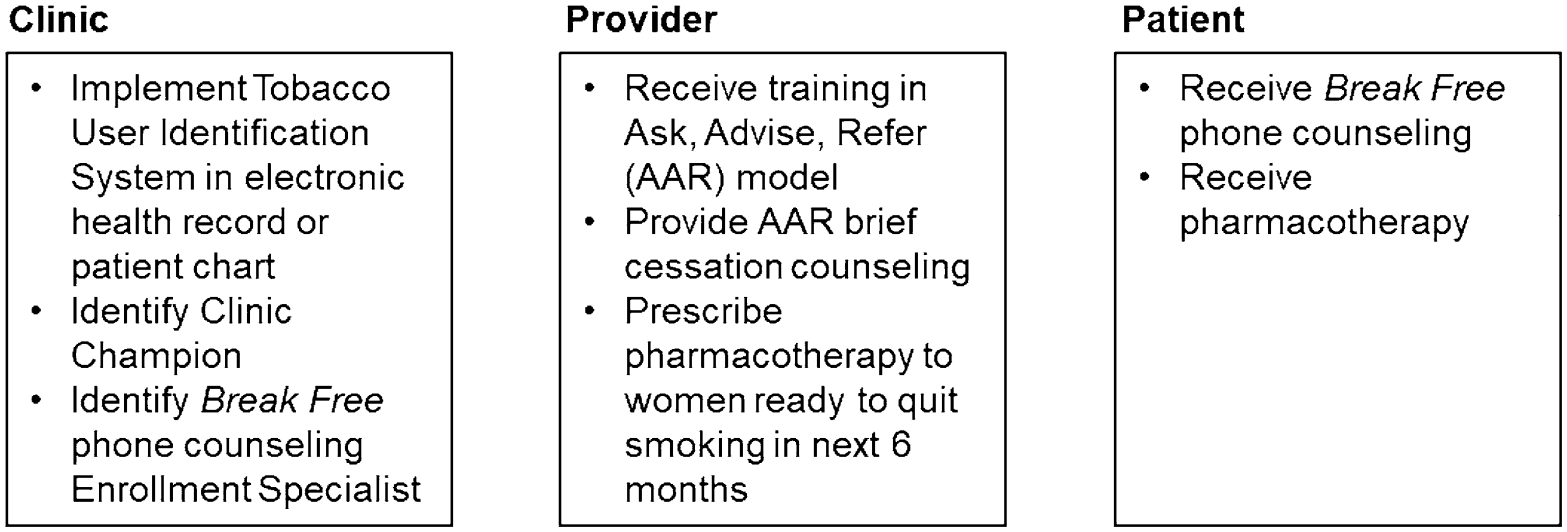
*Break Free* program components

*Break Free* includes clinic, provider, and patient components (Fig. 1). During the year prior to launching *Break Free*, our team conducted community, health system, and clinic assessments; focus groups; and key informant interviews to refine the multilevel intervention components and develop materials. *Break Free* begins in the clinic with brief provider-delivered cessation counseling. During a routine medical visit, providers ask all patients about their tobacco use and document tobacco use and smoking status in the electronic health record. Providers then advise smokers to quit and assess their readiness to quit. Smokers who are ready to quit in the next 6 months receive a prescription for pharmacotherapy and a referral to the *Break Free* Enrollment Specialist. Thus, *Break Free* follows the Ask, Advise, and Refer (AAR) smoking cessation model [23].

#### Clinic components

Each participating clinic is asked to implement a tobacco user identification system [23] in their existing electronic health record that meets the very basic requirement of prompting providers to ask patients about tobacco use. The Survey and Data Collection core will support each clinic to evaluate and update their EHR as need to capture tobacco use information. Each clinic is also encouraged to train at least one employee as a Clinic Champion and a *Break Free* Enrollment Specialist. The Clinic Champion is someone who advocates for the integration of evidence-based smoking cessation treatment into the clinical workflow and keeps clinic staff motivated to promote smoking cessation among their patient population. The Enrollment Specialist is a staff person who acts as a liaison between providers and *Break Free* phone counseling. After a provider refers a patient to *Break Free* phone counseling, the Enrollment Specialist explains the details of the program, distributes patient education materials, and connects the patient with a tobacco treatment specialist (TTS).

#### Provider components

All providers who are directly involved in patient care (e.g., physicians, advanced practice registered nurses) receive a 30–60-min training on how to deliver brief cessation counseling in the clinic [24]. Provider training is delivered via Zoom or Teams (due to the ongoing COVID-19 pandemic; in the future trainings may take place in person) once per intervention phase and is scheduled at a time convenient for the clinic. Providers who are unable to attend the training are offered the recording of the online training. The basic training materials were developed from the Rx for Change© program materials [40] and modified to include additional information about smoking in Appalachia, national data about readiness to quit smoking and use of pharmacotherapy, and other information that is relevant to our theoretical model, the Theory of Planned Behavior [41, 42].

The provider training targets relevant theoretical constructs that are hypothesized to predict providers’ intentions to provide brief cessation counseling to patients using the AAR method. Specifically, content addresses providers’: (1) *normative beliefs* by reviewing smoking prevalence in Appalachia and providing an introduction to the Clinical Practice Guideline; (2) *behavioral beliefs* by describing the efficacy of pharmacotherapy and behavioral counseling; and (3) *perceived behavioral control* by modeling how to implement the AAR model and detailing how to prescribe pharmacotherapy to patients ready to quit smoking. Providers also receive pocket cards (see Additional file 1) that outline *Break Free* phone counseling eligibility criteria and pharmacotherapy options for smoking cessation. Healthcare providers are essential to *Break Free’s* success. They are expected to deliver the AAR model during patient encounters. Thus, providers are to ask about tobacco use, advise users to quit, and refer smokers who are interested in quitting to *Break Free* phone counseling via the Enrollment Specialist.

The *Break Free* Clinic Champions and Enrollment Specialists also attend the provider training to learn the details of the intervention. Following this, Clinic Champions and Enrollment Specialists complete their own 20–30-min training session on Teams or Zoom. This session focuses on the tasks they complete to enroll participants into *Break Free* phone counseling and sustain *Break Free* provider implementation of AAR and TTS-delivered phone counseling in the clinic after the study ends. For example, *Break Free* Enrollment Specialists learn about the content of the educational materials, how to track patient referrals, and how to contact the TTS. Checklists are created for Clinic Champions and *Break Free* Enrollment Specialists to use while working with the *Break Free* program.

Booster sessions are offered to each clinic. Topics include how to better engage smokers in conversations about cessation and how to bill for smoking cessation counseling (this latter topic will be offered during the sustainability phase).

#### Patient components

Smokers enrolled in *Break Free* receive customized patient educational materials, phone counseling, and pharmacotherapy. The patient education materials consist of an informational booklet that describes the benefits of smoking cessation, nicotine withdrawal symptoms, tips for coping with withdrawal and maintaining abstinence, and pharmacotherapy, and a wallet-sized self-monitoring booklet wherein patients record basic information about their smoking behavior (e.g., date, time, circumstances) as they attempt to understand their usage patterns and make quit attempts (see Additional file 1).

To ensure acceptability and health literacy of materials, we conducted focus groups with community members from West Virginia and rural, Appalachian Ohio, Kentucky, and Virginia regions. In each state, two successive focus groups occurred. In the first, participants provided feedback on baseline patient surveys and publicly available smoking cessation materials created by Rx for Change© [40] and the University of Virginia Cancer Center. We used participant feedback to create the customized materials for *Break Free*. In the second focus group, the participants reviewed drafted *Break Free* materials, which were then refined.

Cessation phone counseling is led by a trained TTS. A study-supported TTS is available to all participating clinics during the intervention phase. The TTS is a bachelor’s-level provider who has completed a certified tobacco treatment training program. If a health system has a dedicated TTS, that TTS will be trained in the *Break Free* protocol by the study TTS prior to working with *Break Free* participants. Four counseling calls are included in *Break Free*. During each call, a trained TTS uses motivational interviewing techniques to bolster intention and confidence around smoking cessation, guide smokers through a quit attempt and help them strategize for relapse prevention. The TTS tracks participants’ quit date, smoking cessation outcomes, pharmacotherapy use, and session content. Side effects of pharmacotherapy are monitored during counseling calls. Participants whose side effects are not managed with the usual recommendations (e.g., do not wear an NRT patch at night, rotate NRT patches daily) will be referred to their provider at the clinic. The TTS will call the clinic to notify the provider that the participant is experiencing a side effect that is difficult to manage. If a patient experiences a serious adverse effect in response to pharmacotherapy, that information will be conveyed to the study research team and reported to the IRB.

The *Break Free* delivery model includes a standard counseling program for smokers ready to quit in the next 30 days and a modified counseling program for smokers who are ready to quit in the next 6 months, but not in the next 30 days. *Break Free* offers efficacious strategies [43] regardless of participants’ immediate willingness/readiness to quit (Table 1). The first *Break Free* phone counseling session addresses smoking rate reduction for all participants. Rate reduction occurs by using strategies such as breaking brand loyalty, self-monitoring, and disrupting automatic triggers to smoke. The content of the remaining three phone counseling sessions depends on whether the participant is ready to quit in the next 30 days or not. Session topics and goals are included in Table 1. In summary, the standard program guides a smoker through the quitting process by setting a quit date and creating a relapse prevention plan. The modified program includes goals for reductions in the frequency and amount of smoking between each call.

**Table 1:** *Break Free* protocols for smokers ready to quit immediately and within 6 months

Session #	Standard—quit within 30 days	Rate reduction—Quit within 1–6 months
1	Baseline tobacco use and assessment of tobacco history Rate reduction by 25% Rate reduction strategies, including, situational control (smoke in only certain situations or never smoke in others), temporal control (a time-based strategy), and access (keep cigarettes in an inconvenient spot to avoid “automatic cigarettes”) Nicotine replacement therapy	
2	Set a quit date Prepare to quit	Rate reduction (additional 25%) Discuss future quit
3	Evaluate the quit date Develop short-term relapse prevention plan	Rate reduction (additional 25%; 75% total) Discuss plans short-term relapse prevention for future quit
4	Develop long-term relapse prevention plan	Rate reduction to reduce amount of each cigarette smoked (e.g., marking cigarette with a non-toxic pen to encourage participants to smoke 50% of each cigarette) Discuss longer-term relapse prevention strategy for future quit Encourage targeted future quit date

*Break Free* also includes pharmacotherapy, prescribed by the patient’s provider and in coordination with the TTS. Together, the patient and provider decide which pharmacotherapy is the most appropriate treatment. Options include varenicline, bupropion, or any approved form of nicotine replacement therapy (NRT). Women with health insurance use their benefit to cover pharmacotherapy costs. For uninsured women, we have created a voucher system whereby providers can dispense a voucher for free NRT (gum, patch, or lozenge) or bupropion with the pharmacotherapy prescription. A local pharmacy provides the medication and bills the study using an invoicing system. If varenicline or the nicotine inhaler is chosen, the Enrollment Specialist will complete a Pfizer Inc. application for free medication.

### Hybrid effectiveness-implementation study design

In this cluster randomized trial, health systems are stratified by state and then randomized in a 1:1 ratio to either an Early Arm or Delayed Arm. To reduce the risk for contamination, within each stratum, health systems are randomized into Early Intervention or Delayed Intervention arms. All clinics within each health system are randomized to the same condition (Early or Delayed Intervention). Neither health systems nor study participants are blinded to their intervention assignment.

**Fig. 2:**
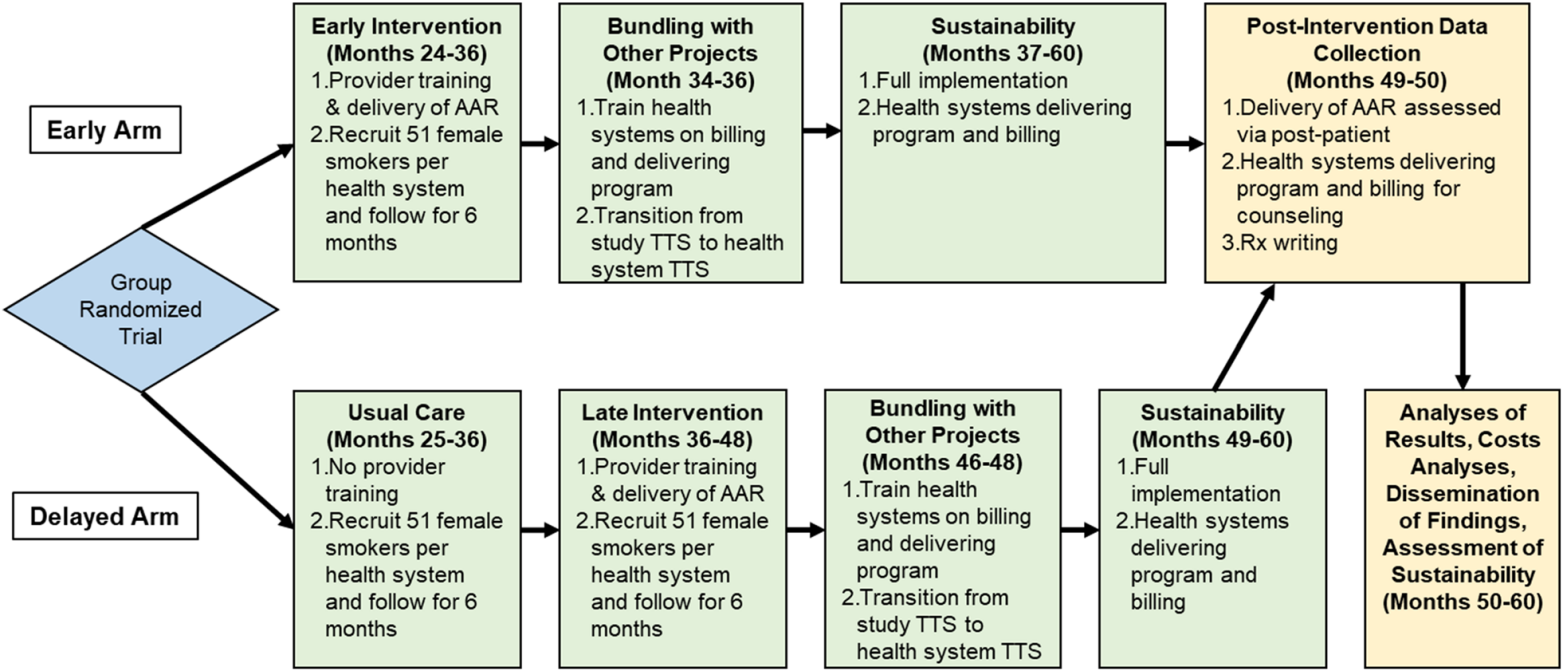
Hybrid implementation-effectiveness study design

Figure 2 outlines the timeline for study implementation and evaluation. Following a planning phase (months 1–23; not shown in the figure), *Break Free* will be implemented in Early Arm health systems over a 12-month period. After this 12-month period ends, research staff will train Early Arm health systems on how to bill for brief counseling (AAR) and smoking cessation phone counseling, as well as how to implement *Break Free* using their own staff and resources as they transition to a full sustainability period. During this period, Early Arm health systems will also learn how to “bundle” all three *Take CARE* projects addressing smoking cessation, HPV vaccination, cervical cancer screening as part of an integrated cervical cancer prevention program disseminated by clinics. Delayed Arm health systems will initially enroll female smokers into an observational study, which will allow estimation of the smoking cessation rate in the target population receiving usual care. This group of women will serve as the comparison for the “effectiveness” of *Break Free*. After 12 months, while the Early Arm health systems are in the sustainability period, Delayed Arm health systems will implement *Break Free*, delivered by our research staff. After that 12-month period ends, they too will then shift to a sustainability period. Implementation of *Break Free* will be evaluated through randomly sampled patient assessments of provider fidelity to AAR and, for women enrolled in *Break Free* TTS counseling, assessments of TTS fidelity to *Break Free* counseling.

#### Early Arm health systems

##### Eligibility criteria

Eligible healthcare systems are those that provide care to female smokers and are in the Appalachian region of the target states. Eligible providers in the healthcare systems are those who are directly involved with patient care (e.g., physicians, advance practice professionals). Eligible patients include women who are: (1) age 18 to 64; (2) smokers who consume at least five cigarettes per day; (3) ready to quit smoking within the next 6 months; (4) English-speaking; (5) able to participate in phone counseling; (6) willing to try pharmacotherapy; and (7) not pregnant.

##### Procedures

The study design procedures of *Break Free* include: (1) training providers, Clinic Champions, and Enrollment Specialists as described above; (2) recruitment and consent of potential participants; (3) provision of cessation counseling and pharmacotherapy; (4) billing for services; (5) fidelity assessments; (6) follow-up assessments; and (7) cost-effectiveness assessments. All study protocols were developed with and reviewed by a Community Advisory Board, External Scientific Advisory Board, and Data and Safety Monitoring Board.

Trainings: As previously described, all providers from participating clinics who are directly involved in patient care will be trained in *Break Free* and the AAR method. Trainings will take place in-person or virtually. The training materials were adapted from Rx for Change^®^, an organization that disseminates public use smoking cessation resources for healthcare providers [40]. In addition, providers will receive pocket cards (see Additional file 1) to remind them of *Break Free* eligibility criteria and pharmacotherapy options for smoking cessation.

All who attend the provider training will be asked to complete a consent form and pre-training assessment. Questions focus on attitudes, confidence, and practices around evidence-based smoking cessation treatment; knowledge of counseling and pharmacotherapy for smoking cessation; and demographics. Immediately after the provider training session, all participants who provided consent and completed the pre-training survey will be asked to complete a post-training survey to assess increased knowledge following the training. Provider surveys will also be distributed at the end of the implementation phase, with similar questions as the baseline survey plus items to assess acceptability and satisfaction with AAR models and *Break Free* specifically. Providers who complete virtual trainings will be sent a link to an online informed consent prior to completing an online survey.

**Fig. 3:**
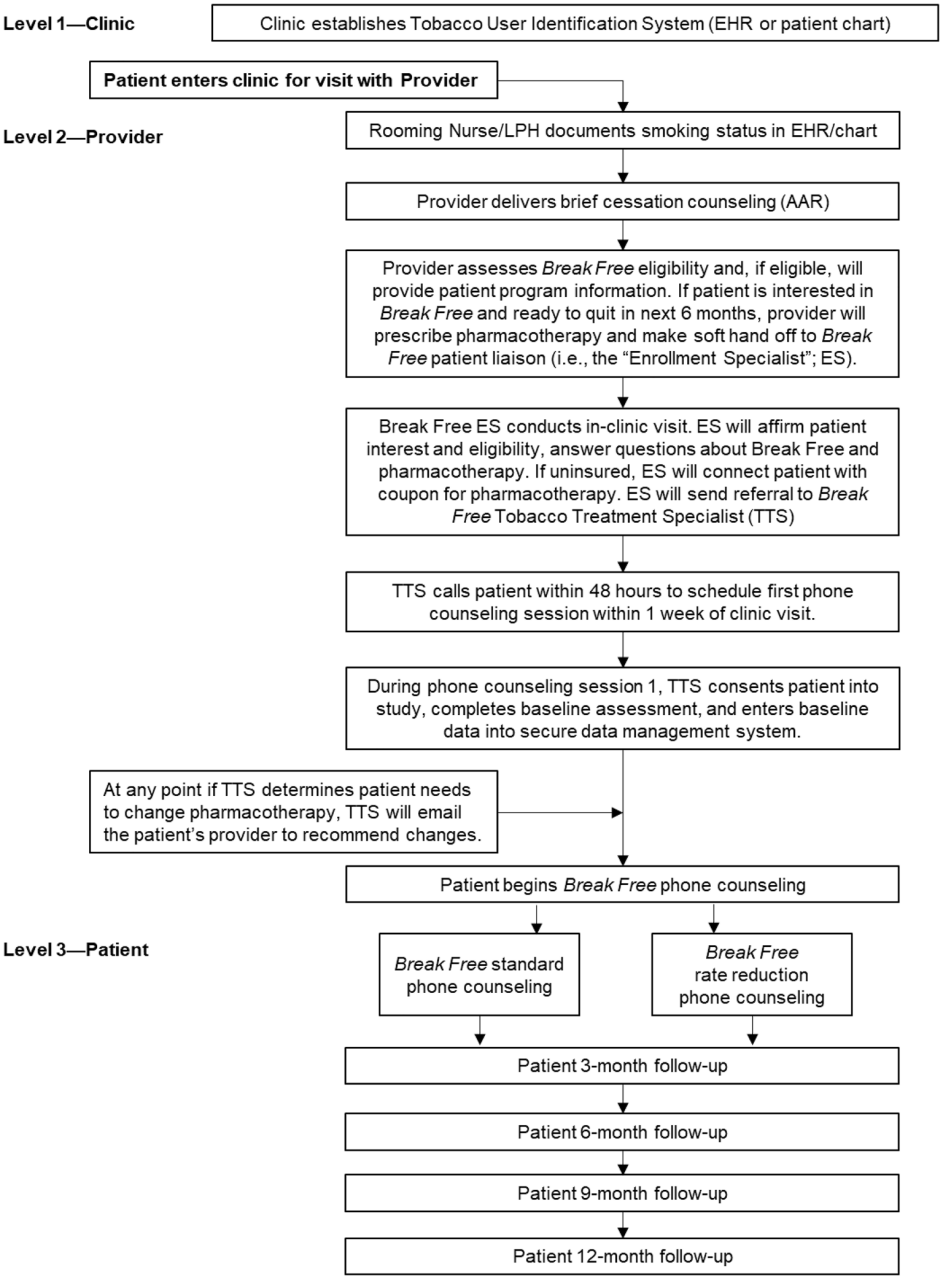
Early Intervention Process Map

Recruitment, Enrollment, and Consent of Participants: Figure 3 outlines the process for participant recruitment and enrollment into the early arm intervention. Eligible patients will be identified during routine clinic visits. Providers will ask patients about tobacco use and advise smokers to quit. Women who are interested in quitting within the next 6 months and are eligible for *Break Free* will receive a prescription for pharmacotherapy and referral to the Enrollment Specialist for that clinic or health system. The Enrollment Specialist will introduce potential participants to the *Break Free* program, distribute the patient educational materials, and collect patient contact information for referral to the study-specific TTS to be consented into the study.

Within a week, the study-specific TTS will contact potential participants and obtain verbal informed consent. If the TTS is unable to reach the potential participant, they will attempt to reach them up to ten times to schedule the informed consent and baseline assessment. Consented participants’ contact information is entered into RedCap data management system for secure storage. Contact information is used to connect participants to *Break Free* phone counseling calls and for follow-up assessments. All participants are assigned a subject ID, which is stored in RedCap independently from participant data. After consent is obtained, the study specific-TTS will administer the baseline assessment via phone. Participants will receive $10 for completing the baseline assessment.

Counseling and pharmacotherapy: The *Break Free* 4-session counseling protocol begins after the baseline assessment is complete. The study-specific TTS will counsel participants from health systems that do not have a dedicated TTS on staff; otherwise, the health system’s TTS will perform the counseling. Again, if the TTS is unable to reach participants for a counseling session, they will call them up to ten times to reschedule. If a participants’ phone number is not working, then the TTS will send a follow-up email and, if needed, a mailed letter, to update participant contact information.

Fidelity assessments: Fidelity to the intervention by providers and the TTS will be assessed separately. To assess provider fidelity to the AAR model, each quarter during a 1-week period, all patients who visit the clinic will be asked to complete an anonymous, self-report, post-provider visit survey. Paper surveys will be collected in a sealed envelope by the check-out staff person at the clinic and mailed to the study team by the Clinic Champion. Patients will also have the option to complete an online survey by scanning a QR code.

To assess TTS fidelity to *Break Free*, ten percent of all *Break Free* participants will be randomly selected and called after a scheduled counseling call to confirm the call occurred and to determine which content was discussed. No financial or other incentives are given for the fidelity assessments.

Patient follow-up assessments: *Break Free* participants will be contacted at 3-, 6-, and 12-months post enrollment to assess current tobacco use and quit attempts via online or phone surveys. Participants will receive a $10 gift card incentive for completion of each follow-up survey. Two weeks prior to each follow-up survey, participants will be sent a proactive reminder email or mailed letter reminding them of the upcoming follow-up assessment.

#### Delayed Arm health systems

While the Early Arm health systems are in the Early Intervention phase, the Delayed Arm health systems will continue usual care (Fig. 1). During this phase, to determine “usual care” tobacco-related outcomes in the Delayed Intervention arms, providers will ask eligible female smokers about their tobacco use and assess readiness to quit. Female smokers who are interested in quitting in the next 6 months will be asked to participate in this observational component of the study. Those who agree to participate will be given either a paper a baseline survey packet with a consent script to sign and the baseline survey in the clinic, where they will be asked to complete it in the clinic and then mail it to the research team (a self-addressed stamped envelope will be included), or an online link with an electronic consent and survey. The consent will include language about follow-up surveys at 3, 6, and 12 months. Study staff will call or email Delayed Arm participants at 3-, 6-, and 12-months post-baseline to assess smoking behaviors. Participants will receive a $10 gift card incentive for completion of each survey. Once Delayed Arm clinics begin the Active Intervention phase, they will follow all procedures implemented in Early Arm health systems as described above.

### Sustainability

Throughout the implementation phase, the study team will periodically meet with health system representatives and clinic managers to develop practical solutions to manage any additional clinical burden. With the transition of the Early Arm health systems to sustainability, the study-specific TTS will no longer provide counseling. Rather, health systems will need to have their own TTS (whether established or newly hired) to deliver counseling to sustain the *Break Free* program. An important component of sustainability will be recovering maximum allowable costs for smoking cessation counseling from health insurers. Thus, providers will be trained on how to bill insurance companies for tobacco cessation counseling. In the sustainability phase, current billing and pharmacotherapy practices of each health system, potential barriers in optimizing reimbursement, and their potential solutions will be documented. The Biostatistics and Evaluation core will assist in data extraction for these measures.

Prior to the sustainability period, the other two interventions that are part of *Take CARE* (addressing HPV vaccination and cervical cancer screening) will be “bundled” with the *Break Free* smoking cessation intervention as part of a multifaceted cervical cancer prevention program. We will provide a refresher training for all clinic staff and providers that reviews how to implement all three interventions with patients. We will also train clinic staff and providers on how to bill for services provided specific to all three interventions.

### Program evaluation

*Take CARE* program evaluation will include documentation and monitoring of implementation (process evaluation), assessment of progress in reaching goals and objectives (outcome evaluation), and the extent to which interventions are adopted by clinics and sustained over time (sustainability evaluation). Consequently, the Survey and Data Collection and Biostatistics and Evaluation cores will support evaluation for each of the three *Take CARE* initiatives separately as well as the *Take CARE* program overall. Evaluation at the overarching program level, will investigate the relative contributions of individual, community, clinic, and intervention effects on uptake of the three *Take CARE* initiatives by participating clinics. For this study protocol, we present detailed information on the *Break Free* project evaluation, including measures, sample parameters, and statistical analyses.

### Process, outcome, and sustainability measures

#### Process evaluation

##### Provider outcomes

*A*cceptability of the *Break Free* intervention will be examined by estimating adoption by providers and staff. These provider-level outcomes will include self-reported changes in the delivery of AAR and the rate of referrals to the in-clinic TTS.

##### Patient outcomes

Fidelity to the intervention will be assessed two ways. First, 10% of women will be randomly selected and called by a study staff person the day after *Break Free* cessation phone counseling and asked standardized questions about the content of the call. Second, self-administered, anonymous patient post-visit surveys will be completed throughout the implementation phase. These fidelity surveys will ask patients to report whether the provider: (1) asked about tobacco use; and if a smoker, (2) advised the individual to quit; (3) discussed cessation; and (4) referred the smoker to counseling. The proportions of patients being asked, advised, and referred are the outcomes.

#### Outcome evaluation

##### Provider outcomes

The primary outcome among providers is adoption of the AAR components, measured by self-report on the provider survey administered at the end of the implementation phase. We will also assess responses to the knowledge items on the survey (pre vs. post-training, and follow-up).

##### Patient outcomes

The primary outcomes among *Break Free* patient participants include self-reported: (1) 7-day point prevalence abstinence; (2) 7-day floating abstinence during any period since last assessment; (3) prolonged abstinence (no smoking after a 2-week grace period after the quit date); and (4) at least one 24-h quit attempt. A 24-h quit attempt is an important endpoint given that it is associated with a greater likelihood of future cessation [39]. Secondary outcomes among *Break Free* participants include use of pharmacotherapy and number of counseling sessions completed.

#### Sustainability evaluation

*Sustainability* will be measured by: (1) documentation of patient tobacco use status in the EHR; (2) TTS self-report of continued smoking cessation counseling; and (3) billing-related documentation of number of counseling sessions billed for overall and for each smoker who has at least one *Break Free* counseling session. The Survey and Data Collection core will support EHR and billing data extraction. With the support of the Biostatistics and Evaluation core, we will also assess cost-effectiveness by considering costs of each component including pharmacotherapy, clinic counselor time and training, smoking cessation counselor time and training, telephone and material costs, and other administrative costs.

### Sample size and power analysis

The sample size for the primary outcome (i.e., smoking cessation among female smokers) is 51 female smokers per health system, for a total of 510 participants. This sample size is based on a two-sample test of the 7-day point prevalence abstinence at 6 months with power calculated using a standard cluster randomized design formula [44]. Assuming a 10% quit rate in the delayed group, a 25% quit rate in the early intervention group, and a conservative estimate of the intraclass correlation of 0.017, a total of 10 health systems, equally randomized to Early and Delayed Intervention groups, a total of 43 smokers per health system will provide 80% power at a two-sided significance level of 5%. The sample size was inflated to 51 smokers per health system (or 510 total) to account for an estimated 20% dropout.

### Statistical analyses

#### Missing data

We recognize that despite efforts to minimize dropout and missing data, there will be subjects who miss assessments or drop out of the project prior to completion. To avoid biases due to relationships between dropout and patient characteristics, we will use multiple imputation methods appropriate for multilevel data [45] to impute missing outcomes. Imputation models will include health system, intervention group, and any patient factors that differ between those who dropped out and those completing the study. The number of imputed data sets will equal the dropout percentage as recommended by White and colleagues [46]. Results from the imputed data sets will be combined using Rubin’s rules [47].

#### Provider outcomes

For all provider outcomes, we will conduct intent-to-treat analyses (ITT), such that all participants who are randomized will be included in statistical analyses.

Mixed logistic regression models, with random effects for provider and clinic, will be used to assess the proportion of patients receiving AAR over the time periods pre-intervention, during intervention and in the post-intervention periods.

Mixed models will be used to assess measures of staff and provider satisfaction with the program, using random effects for health system. Subsequent analyses will add provider characteristics in order to evaluate whether satisfaction with *Break Free* differs by these characteristics. Mixed models will be used to evaluate changes in provider knowledge and attitudes over time, using random effects for health system.

#### Patient outcomes

For all patient outcomes, we will conduct ITT analyses. We will assume that non-responders to surveys will be continued smokers.

Hierarchical (mixed) models will be used to compare smoking cessation outcomes at 6- and 12-months between female smokers in the Early and Delayed Arm health systems. Logistic models will be used for the binary outcomes, including the point prevalence, floating and prolonged abstinence, and at least one 24-h quit attempt. Subsequent analyses will adjust for patient-level characteristics in comparing smoking cessation at 6 and 12 months among patients in the Early and Delayed Arm health systems.

Mixed models will be used to assess measures of participant satisfaction with *Break Free*, using random effects for health system and primary provider. Subsequent analyses will add patient characteristics in order to evaluate whether satisfaction with the *Break Free* program differs by these characteristics.

#### Cost-effectiveness

A cost-effectiveness analysis will be performed at the end of the implementation phases. Costs of each component of *Break Free* will be tracked, including pharmacotherapy, patient liaison time and training, TTS time and training, telephone and material costs, incentives, and other administrative costs. We will value the costs of each activity using standard costs. Cost-per-quit estimates will be calculated.

#### Sustainability

We will track the rate at which women are referred to counseling and the billing for cessation counseling. Initial analyses will use hierarchical models for count data, more specifically, negative binomial regression models, to estimate the trends over time. Subsequent analyses will use ‘interrupted time series analysis’ [48] to track the changes in rates of referrals and billing for smoking cessation over time. These analyses will estimate the effect of policy changes at the health system and health center levels on these rates, accounting for the longitudinal design and for potential seasonal cycles in referrals and smoking cessation counseling.

### Data management and monitoring

Outcome measures (smoking-related, provider, sustainability) will be obtained from self-administered patient surveys (in clinic, online) and TTS-delivered phone assessments, provider surveys (in clinic, online), and review of medical charts using electronic health records. We will use REDCap as our data entry system for these data.

A Data and Safety Monitoring Board (DSMB) for the overarching *Take CARE* program will review protocol and monitor study progress and outcomes, make recommendations, and ensure that data and human safety requirements are met. The DSMB is comprised at least five individuals, including clinicians and researchers experienced in implementation science, clinical trials, and cervical cancer; a clinical biostatistician, and a layperson patient advocate. No members of the DSMB is associated with the three initiatives that comprise the overall *Take CARE* research program.

## Discussion

*Break Free* is one component of a comprehensive, integrated program to reduce the burden of cervical cancer incidence and mortality in Appalachia through interventions delivered in diverse primary care clinics across four states. The broader program, *Take CARE,* focuses on rural Appalachia, an area with high cervical cancer burden [3, 4] and significant barriers to accessing traditionally provided cervical cancer preventive services [8, 9, 16, 18, 49].

A significant strength of *Break Free* is that the program is easily adapted to unique clinic settings and varying available resources. For example, in smaller clinics with fewer staff, there may not be adequate staffing to establish a *Break Free* Enrollment Specialist to meet with patients after provider referral. In these cases, patients may be referred directly by the provider to the TTS. Successful implementation of *Break Fre*e in rural Appalachian health systems has potential to create a sustained impact on health at the population level by improving rural patients’ access to high quality, culturally sensitive, and locally sourced evidence-based smoking cessation treatment. By the end of this study, staff and providers in each health system will be trained in how to deliver and bill for brief provider-delivered cessation counseling (i.e., AAR). Health systems will also have their own TTS trained to deliver *Break Free* phone counseling plus pharmacotherapy to their patients. If successfully shown to be sustainable, *Break Free* could be disseminated widely in clinics within Appalachia to any and all patients who smoke, across heterogeneous levels of readiness to quit, as well as to healthcare systems in other underserved geographic settings or patient populations.

In this study, we applied *Break Free*, a clinic-based smoking cessation program, to reduce cervical cancer risk among women in Appalachia; however, because smoking is a leading cause of chronic disease and cancer morbidity [22], *Break Free* has the potential to impact chronic disease and cancer outcomes beyond cervical cancer. This is especially salient for Appalachia where rates of tobacco-related disease mortality, including cancer, heart disease, and chronic obstructive pulmonary disease [50] are disparately high compared to the US population. Alarmingly, the declines in cancer and heart disease mortality rates observed across the US over the past decade are less evident in Appalachia [50]. Increasing tobacco cessation among Appalachian residents, and especially those in rural areas, could substantially reduce chronic disease incidence and mortality in this population with high health disparity. Future research might extend assessment of effectiveness of *Break Free* to other groups such as Appalachian men who smoke [14], or with other high-risk Appalachian populations (e.g., lesbian, gay, and bisexual people [51–54]) wherein smoking is a persistent disparity.

## Limitations

Despite efforts to minimize bias, there are limitations to this planned study. First, results may be subject to observation bias, as clinics in this cluster randomized trial are unblinded after baseline assessment. Second, smoking behavior and cessation outcomes are measured by self-report, and participants’ responses may be influenced by social desirability. Self-report measures are a practical compromise. While biochemical validation or data from EHRs documenting smoking status would be considered a “gold standard” for tobacco studies, neither are especially practical for researchers or partner clinics. The *Break Free* study is designed to assess the effectiveness of a well-established intervention that combines behavioral counseling with pharmacotherapy. Also, biochemical verification of smoking status is not always needed in pragmatic randomized control trials [55] and has lower response rates than self-report data [56]. Third, missing data can be an issue in trials with longitudinal designs; however, the planned 6- and 12-month follow-up is consistent with current recommendations for smoking cessation studies [56] and the planned analyses will follow intent-to-treat methods to minimize bias from dropout over time. Finally, beyond collecting information about each clinic’s specific EHR, including any existing processes to identify tobacco users, clinic-level data on other variables that may affect implementation of *Break Free* (e.g., smoking rates of clinic providers and staff, access to smoking cessation for clinic employees) will not be collected in this study.

## Generalizability

This study assesses the effectiveness and implementation of a smoking cessation program delivered in primary care clinics across four Appalachian states. While Appalachia is a unique geographic and cultural region, its residents experience barriers to smoking cessation—including pro-tobacco norms, low provider knowledge and self-efficacy, minimal access to evidence-based counseling and affordable pharmacotherapy—that are also evident in non-Appalachian, rural geographic regions in developed countries [57–59]. The provider training program uses public use educational materials and the smoking cessation protocol is based on the recommendations included in the Clinical Practice Guideline [23]. To this end, it is possible the planned protocol and results from *Break Free* may be applied to develop and test multilevel, clinic-based smoking cessation interventions in other rural regions and countries other than the US (Fig. 3).

## Conclusion

Smoking rates in Appalachian regions are among the highest in the US and because smoking continues to decline in urban regions, rural-to-urban disparities are widening [60]. The *Clinical Practice Guidelines* recommend that healthcare systems adopt institution-wide changes to promote smoking abstinence among patients [23]. *Break Free* is designed to assess the effectiveness and implementation of three *Clinical Practice Guideline* recommendations to implement: (1) a tobacco user identification system at the clinic level; (2) provider-delivered brief counseling plus referral to supportive cessation services; and (3) a smoking cessation program that includes behavioral counseling and pharmacotherapy. The overall goal is to decrease cervical cancer morbidity and mortality in Appalachia by increasing tobacco cessation among women who smoke, a high-risk population for cervical cancer, as part of a larger program to address the elevated rates of cervical cancer in Appalachia.

## Contributions to the literature

- This protocol describes *Break Free*, Project 1 of *Take CARE* (P01 CA229143), an integrated cervical cancer prevention program designed to address three modifiable risk factors for cervical cancer in Appalachia: cigarette smoking, low rates of HPV vaccination, and low rates of cervical cancer screening.
- This protocol is an example of a hybrid effectiveness implementation study design for a tobacco cessation program implemented into healthcare clinics in Appalachia, where both smoking rates and cervical cancer rates are high.
- The *Break Free* program offers clinics in Appalachia an integrated process for supporting goals of reducing smoking rates among its patients via office system strategies to identify current tobacco users, disseminate evidence-based smoking cessation treatment, and provide clinics with tools to support the sustainability of the program.
- This study protocol uses the implementation science framework and a cluster randomized trial study design to examine (1) effectiveness of *Break Free*, (2) provider and patient satisfaction with *Break Free*, and (3) sustainability of *Break Free*.

## Supplementary Information

**Additional file 1.** *Break Free* Educational Print Materials

## Data Availability

Not applicable.

## Abbreviations

AAR: Ask, Advise, Refer
CARE: Clinical Avenues to Reach Health Equity
DSMB: Data and Safety Monitoring Board
EHR: Electronic Health Record
IRB: Institutional Review Board
ITT: Intent-to-treat
NRT: Nicotine replacement therapy
TTS: Tobacco treatment specialist
US: United States
