# Correction to: A cluster randomized controlled trial for a multi-level, clinic-based smoking cessation program with women in Appalachian communities: study protocol for the “Break Free” program

**DOI:** 10.1186/s13722-022-00304-7

**Published:** 2022-04-01

**Authors:** Joanne G. Patterson, Tia N. Borger, Jessica L. Burris, Mark Conaway, Robert Klesges, Amie Ashcraft, Lindsay Hauser, Connie Clark, Lauren Wright, Sarah Cooper, Merry C. Smith, Mark Dignan, Stephenie Kennedy-Rea, Electra D. Paskett, Roger Anderson, Amy K. Ferketich

**Affiliations:** 1 Division of Epidemiology, College of Public Health, The Ohio State University, 354 Cunz Hall, 1841 Neil Avenue, Columbus, OH 43210 USA; 2 The Ohio State University Comprehensive Cancer Center, Columbus, OH USA; 3 Department of Psychology, University of Kentucky, Lexington, KY USA; 4 Department of Public Health Sciences, University of Virginia, Charlottesville, VA USA; 5 West Virginia University, Morgantown, WV USA; 6 UVA Cancer Center, University of Virginia, Charlottesville, VA USA; 7 College of Public Health, The Ohio State University, Columbus, OH USA; 8 Department of Internal Medicine, College of Medicine, University of Kentucky, Lexington, KY USA; 9 Markey Cancer Center, University of Kentucky, Lexington, KY USA; 10 West Virginia University Cancer Institute, Morgantown, WV USA; 11 Department of Medicine, School of Medicine, West Virginia University, Morgantown, WV USA; 12 Division of Cancer Prevention and Control, Department of Internal Medicine, College of Medicine, The Ohio State University, Columbus, OH USA; 13 School of Medicine, University of Virginia, Charlottesville, VA USA

## Correction to: Addict Sci Clin Pract (2022) 17:11 https://doi.org/10.1186/s13722-022-00295-5

Following publication of the original article [1], minor errors were found in Fig. 2, the corrected figure is provided below:
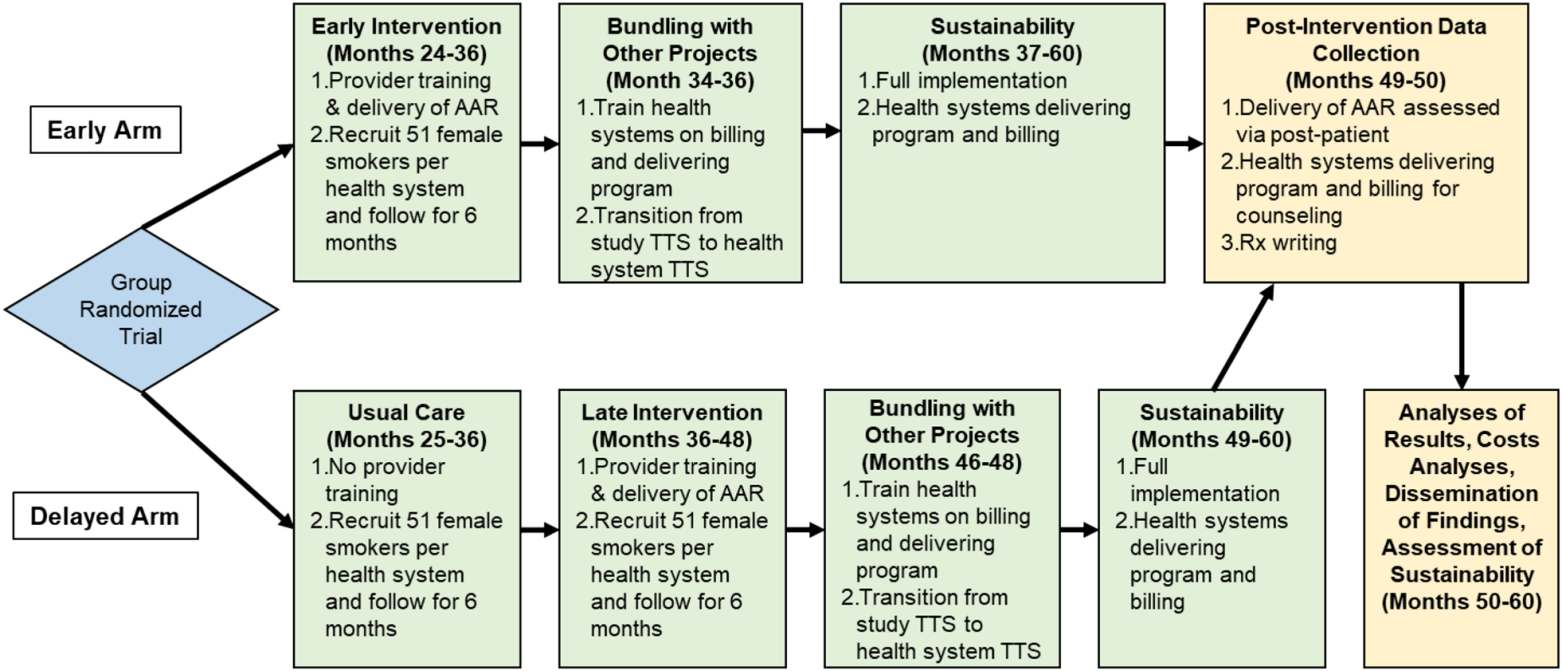


The original paper has been updated.
