# Supplementary material for: A cluster randomized controlled trial for a multi-level, clinic-based smoking cessation program with women in Appalachian communities: study protocol for the “Break Free” program
Source: Addict Sci Clin Pract. 2022 Feb 14;17:11. doi: 10.1186/s13722-022-00295-5 (PMC8842942; doi:10.1186/s13722-022-00295-5)

**Additional file**

[*Break Free* Provider Informational 1-pager 2](#_Toc63774019)

[*Break Free* Provider Pocket Card 3](#_Toc63774020)

[*Break Free* Patient Education Booklet 4](#_Toc63774021)

[*Break Free* Patient Self-Monitor Booklet 8](#_Toc63774022)

# *Break Free* Provider Informational 1-pager

This informational 1-page flyer was designed to be displayed in patient visit rooms.

*
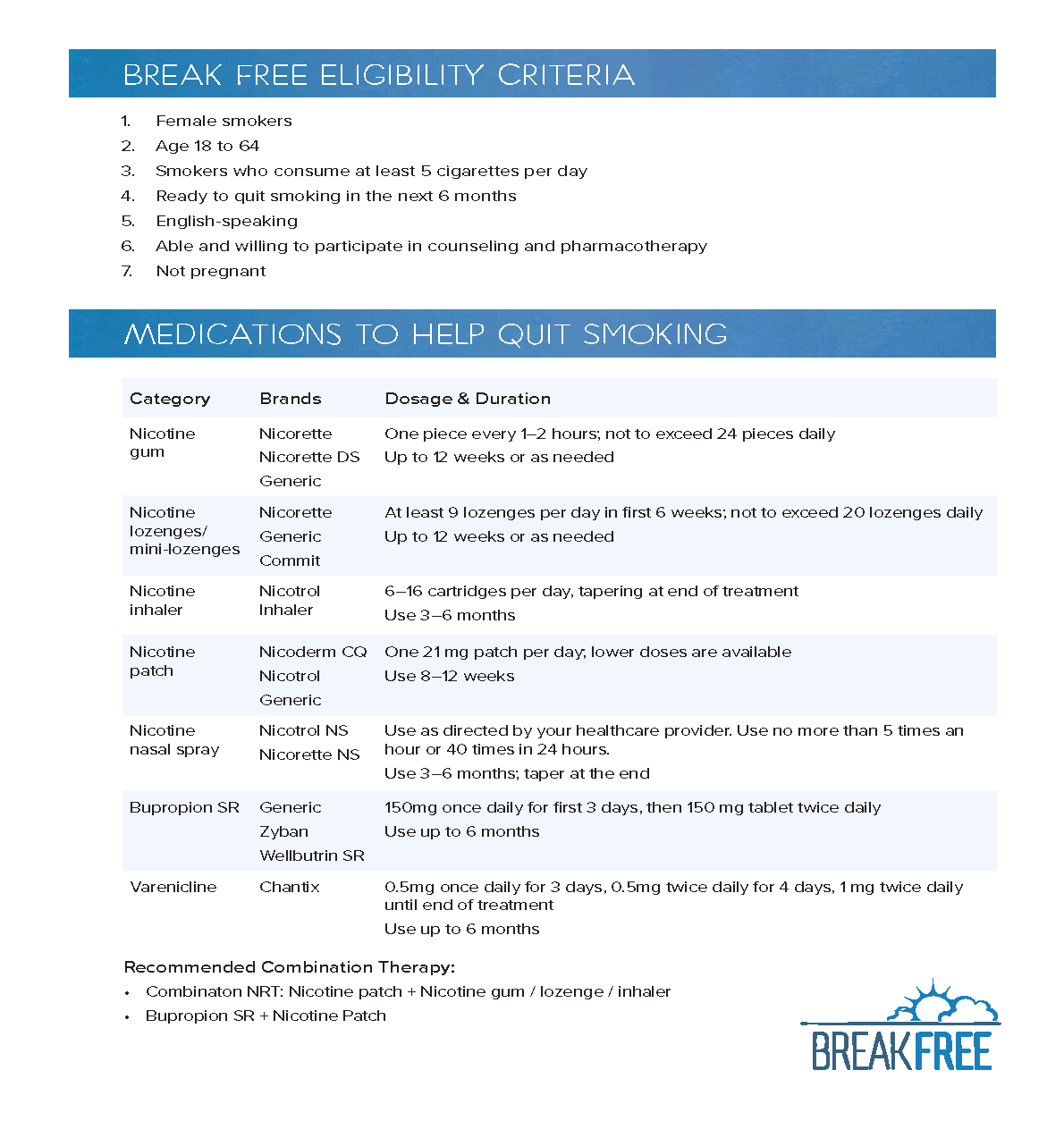
*

# *Break Free* Provider Pocket Card

This informational card was laminated and designed to fit in providers’ pockets.

FRONT BACK


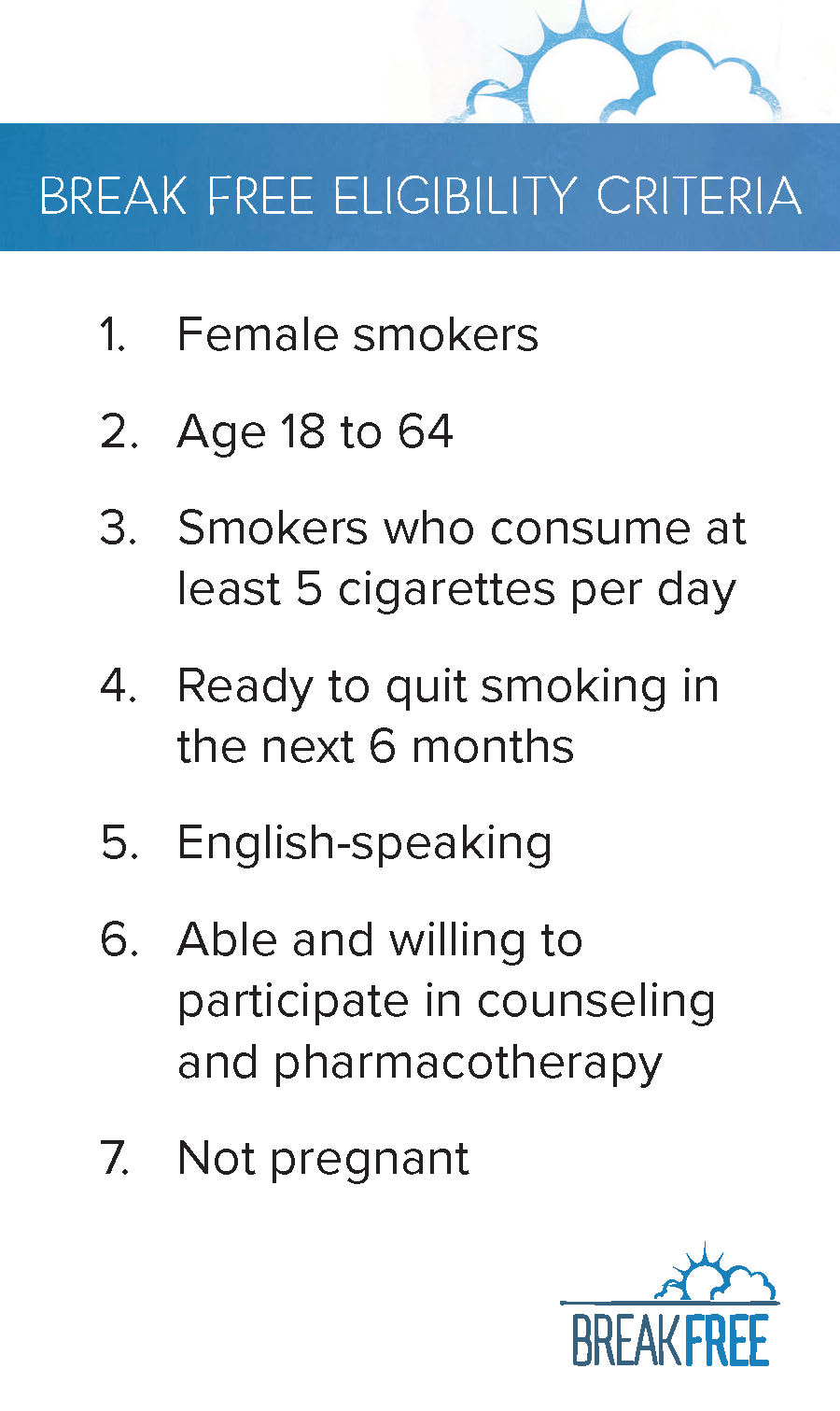

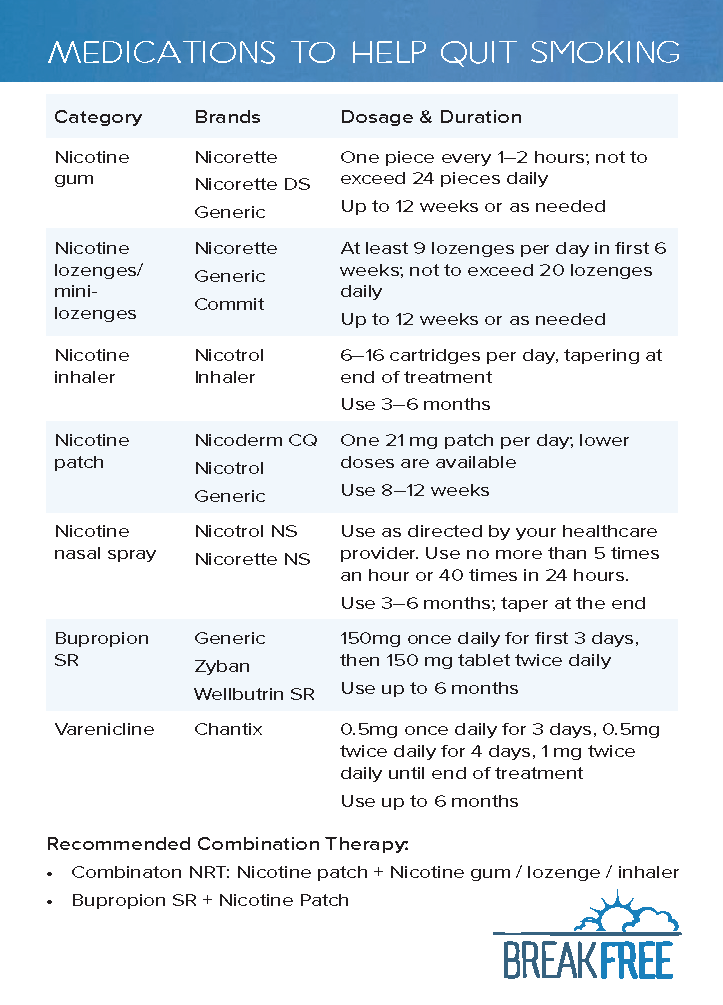


# *Break Free* Patient Education Booklet

This informational booklet is given to patients by the *Break Free* Enrollment Specialist upon referral to the *Break Free* program.

*Pages 1 and 2*

*
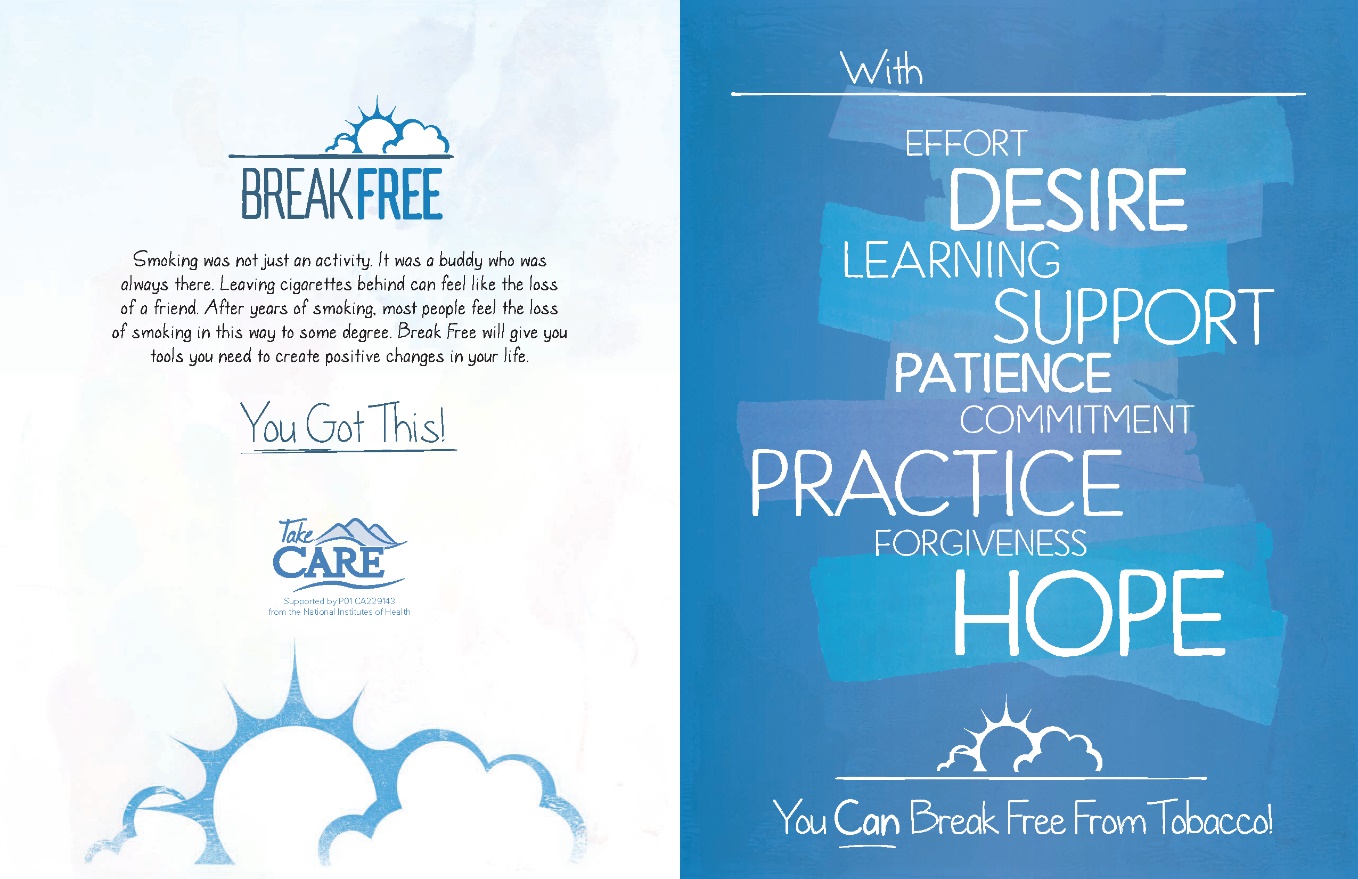
*

*Pages 3 and 4*

*
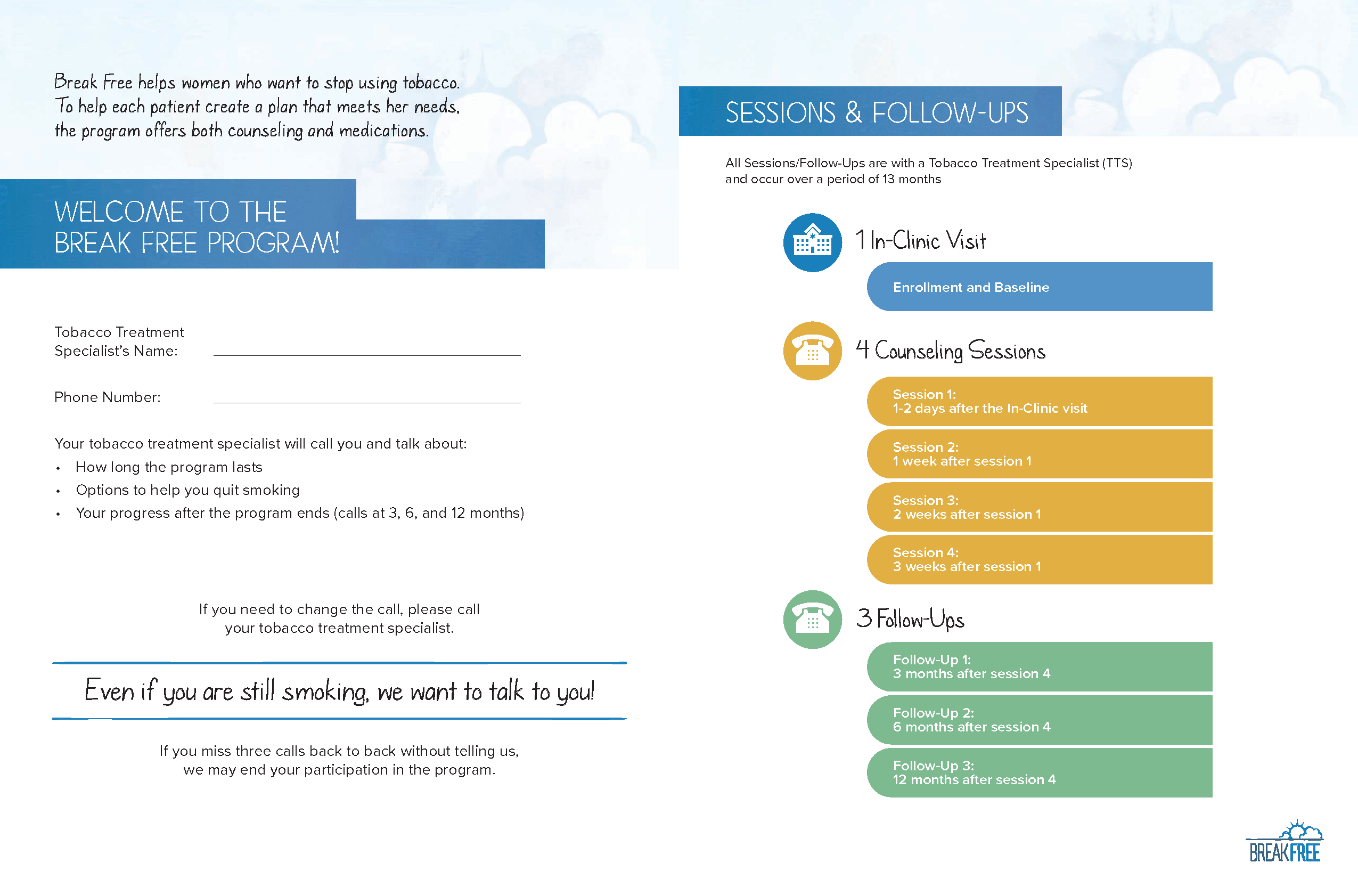
*

*Pages 5 and 6*

*
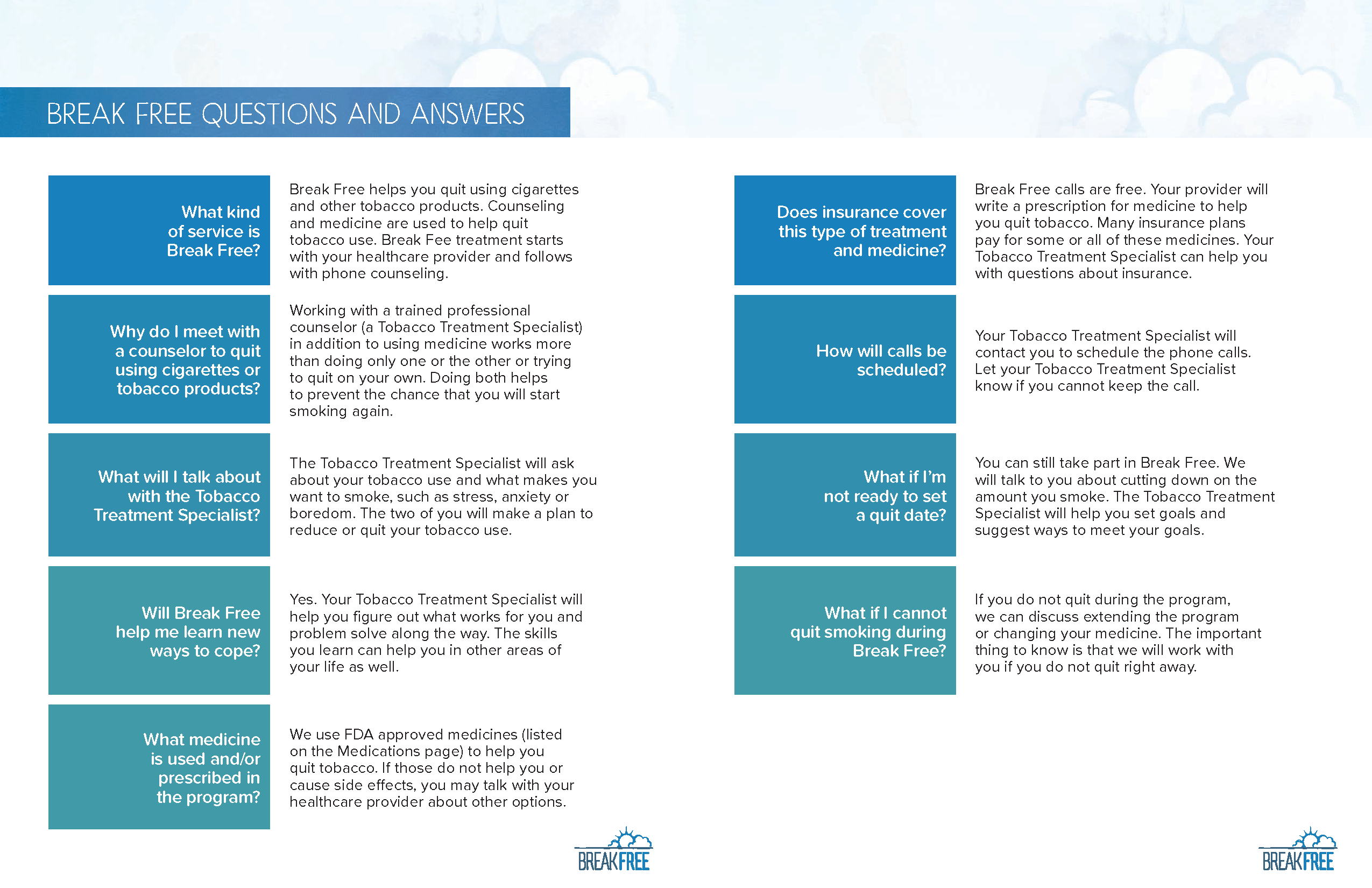
*

*Pages 7 and 8*

*
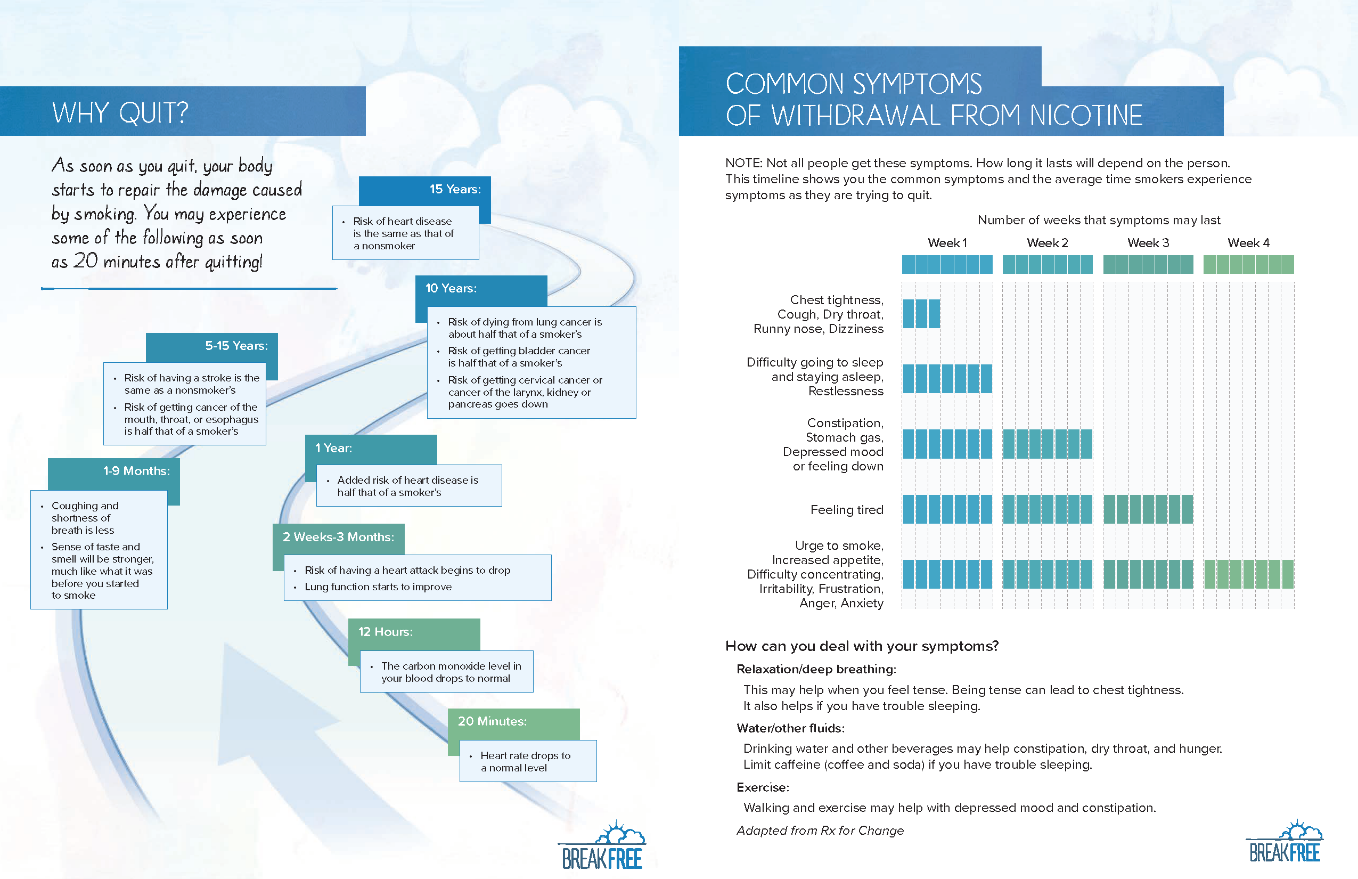
*

*Pages 9 and 10*

*
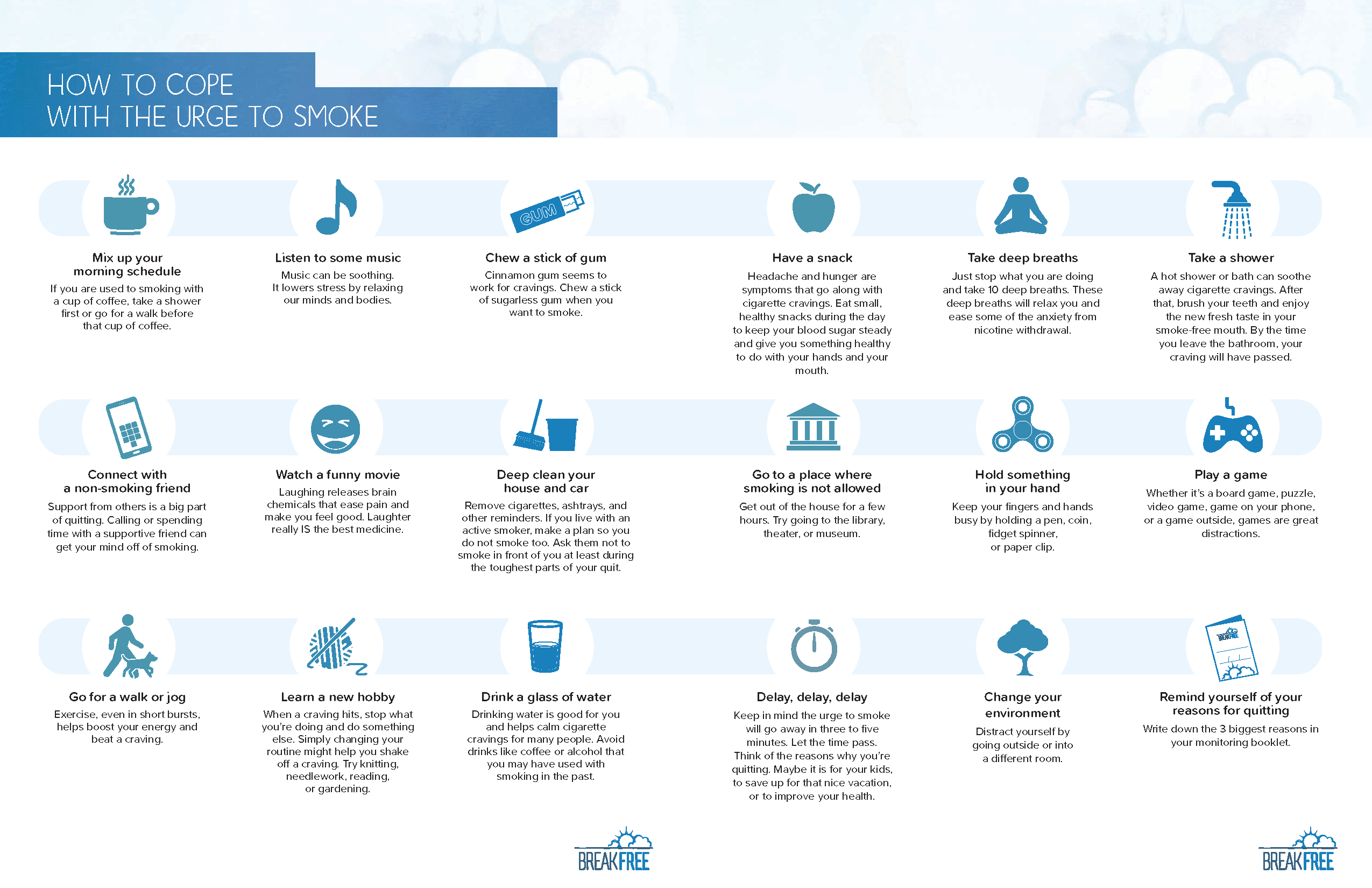
*

*Pages 11 and 12*

*
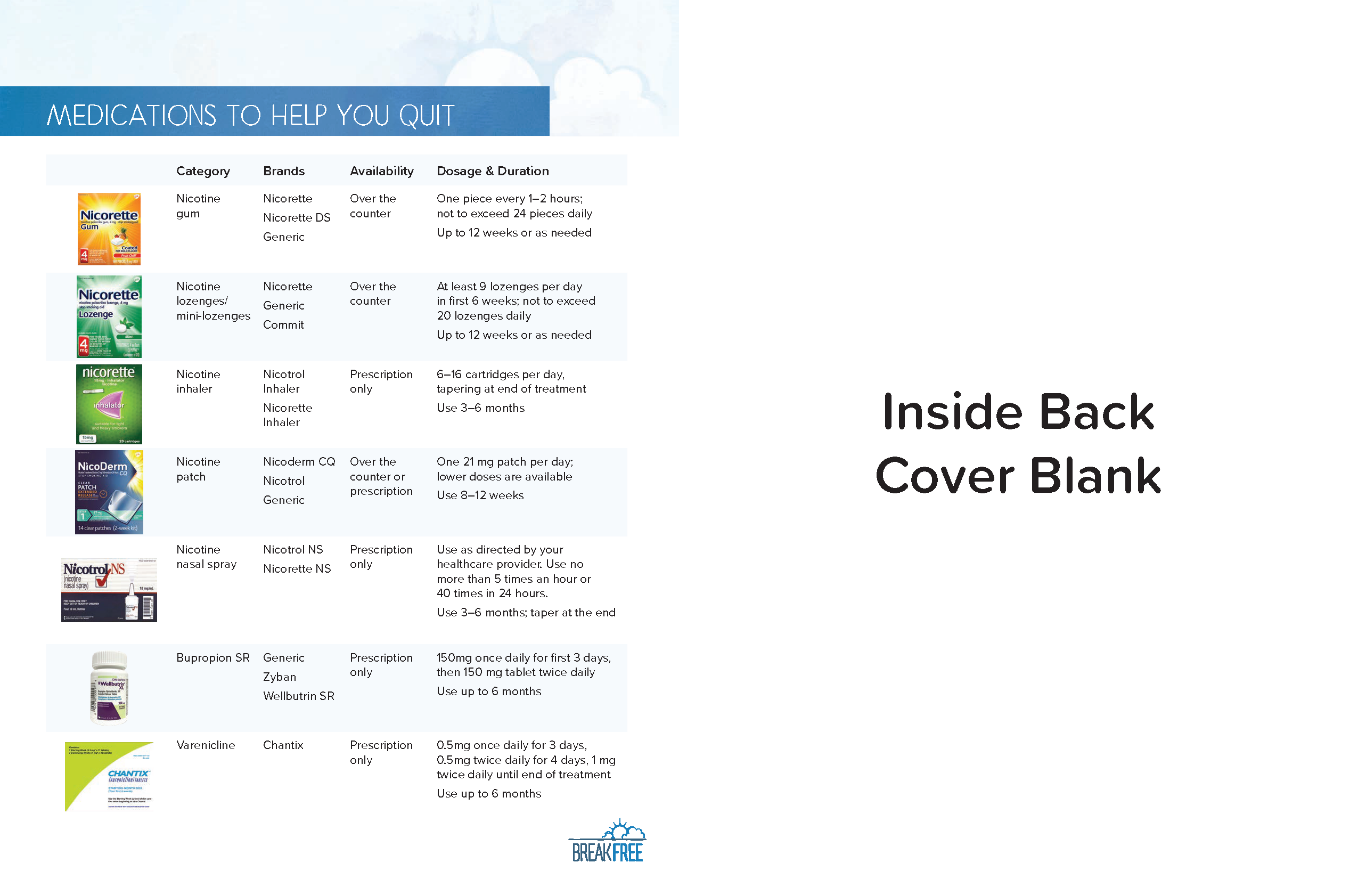
*

# *Break Free* Patient Self-Monitor Booklet

This pocket-sized booklet is designed to help patients monitor their smoking habits. It is given to patients by the *Break Free* Enrollment Specialist upon referral to the *Break Free* program.

*Back and Front*


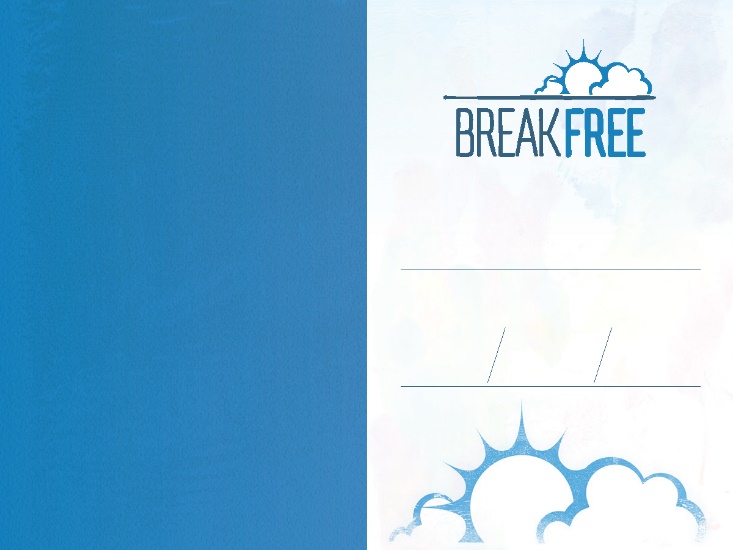


*Pages 1 and 2*


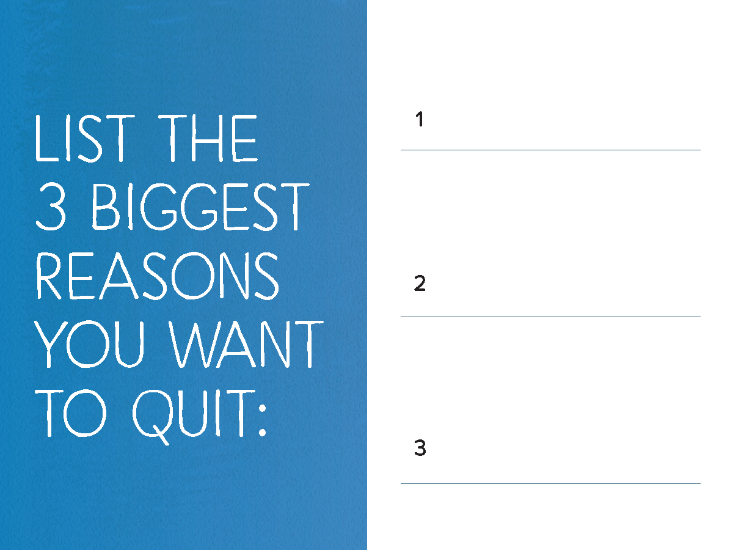


Pages 3 and 4


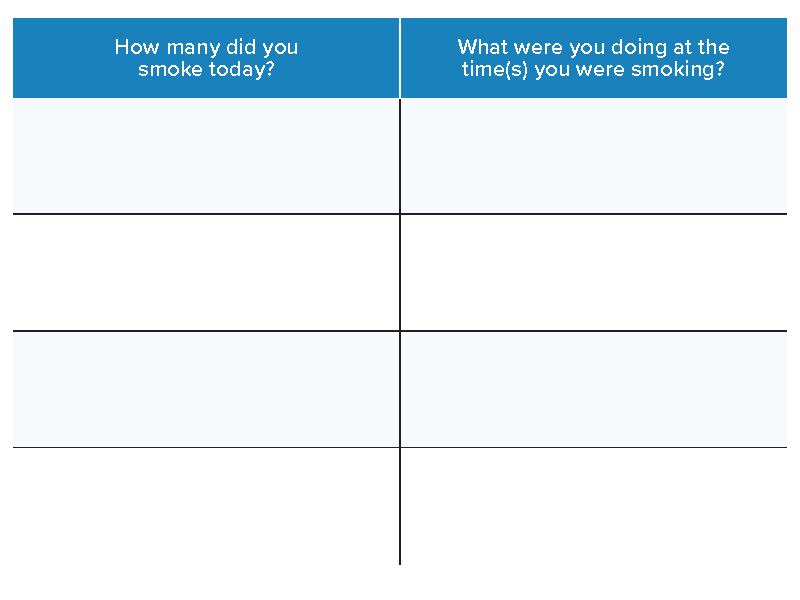

Supplement: Supplementary file 1 — Additional file 1. Break Free Educational Print Materials [file 13722_2022_295_MOESM1_ESM.docx]
